# Comprehensive evaluation of the heat tolerance of non-heading Chinese cabbage based on multifactorial statistical analysis

**DOI:** 10.3389/fpls.2026.1860156

**Published:** 2026-07-07

**Authors:** Mengting Xiao, Zhanyuan Gao, Shiling Meng, Ying Li, Changwei Zhang, Xilin Hou

**Affiliations:** 1 National Key Laboratory of Crop Genetics & Germplasm Innovation and Utilization, Key Laboratory of Biology and Genetic Improvement of Horticultural Crops (East China), Ministry of Agriculture and Rural Affairs of China, Engineering Research Center of Germplasm Enhancement and Utilization of Horticultural Crops, Ministry of Education of China, Nanjing Agricultural University, Nanjing, China; 2 Nanjing Tianliang Bioengineering Technology Co., Ltd., Nanjing, China

**Keywords:** comprehensive evaluation, heat tolerance, high-temperature stress, non-heading Chinese cabbage, principal component analysis

## Abstract

Non-heading Chinese cabbage is a cool-season crop, and high temperature has become a key factor limiting its quality and yield. Given that plant heat tolerance is a complex quantitative trait regulated by multiple genes, establishing a comprehensive evaluation system integrating multiple physiological and biochemical indicators is of great significance. In this study, 35 varieties of non-heading Chinese cabbage germplasm were used to investigate heat damage indices (HDI) and measure physiological and biochemical indicators under summer high-temperature stress, aiming to provide a basis for heat tolerance evaluation. Correlation analysis revealed significant correlations among the physiological and biochemical indicators, indicating information overlap. Principal component analysis (PCA) was subsequently employed to extract six independent composite indicators. A composite index of heat tolerance productivity, namely the Heat Tolerance Productivity Index (HTPI), was obtained through membership function analysis, and cluster analysis classified the tested germplasm into four heat tolerance levels. A regression equation for evaluating heat tolerance in non-heading Chinese cabbage was successfully established. Eleven key heat tolerance indicators were identified, and two highly heat-tolerant varieties, B21 and B32, with excellent comprehensive traits were selected. The comprehensive evaluation system established in this study not only provides an effective tool for high-throughput screening of heat-tolerant germplasm resources but also lays a solid foundation for subsequent genetic improvement and molecular breeding of heat-tolerant varieties. However, this study was conducted only at the seedling stage, and did not evaluate heat tolerance during the more sensitive reproductive stages (flowering and bolting).

## Graphical Abstract

**Figure:**
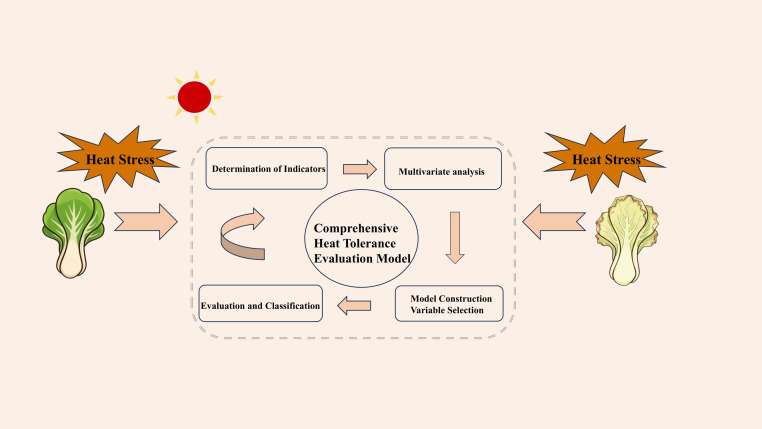

## 1 Introduction

Non-heading Chinese cabbage (*Brassica campestris* ssp. *chinensis* Makino) is native to China and belongs to the genus *Brassica* in the family Brassicaceae (Dai et al., 2021). Owing to its long cultivation history, high nutritional value, short growth cycle, and ease of cultivation, it is widely grown in the Yangtze River Basin and southern China, accounting for approximately 30% of the total vegetable cropland area in cities along the middle and lower reaches of the Yangtze River (Zhang et al., 2020).his leafy vegetable is extensively cultivated and highly popular worldwide, playing a significant role in ensuring a balanced vegetable supply (Li et al., 2020).

Non-heading Chinese cabbage prefers cool and temperate conditions, with an optimal growth temperature of 18-25°C. In recent years, rising temperatures have significantly affected its growth and development, and both yield and quality decline markedly when ambient temperatures exceed 25°C. Consequently, high temperature has become a key factor limiting the quality and yield of non-heading Chinese cabbage (Liu et al., 2018; Tian et al., 2024). Therefore, breeding for heat tolerance is of particular importance. There is an urgent need to screen varieties with strong summer survival capacity to withstand high-temperature stress, thereby improving yield and quality, ensuring a year-round vegetable supply, and safeguarding food security.

Previous studies have shown that high-temperature stress can lead to reduced seed viability, inhibited pollen germination, slowed root and stem growth, induce leaf senescence and abscission, and cause grain shriveling (Wahid et al., 2007; Bahuguna and Jagadish, 2015; Sage et al., 2015). Under high-temperature conditions, cabbage exhibits slow growth, wilting, and leaf yellowing; in severe cases, these symptoms lead to poor quality, increased pathogen susceptibility, and reduced yield (Yue et al., 2021). As the greenhouse effect intensifies, plants are increasingly exposed to temperature stress, making summer survival a pressing concern. In this context, a key determinant of successful summer survival is the strength of heat tolerance.

Under heat stress, plants accumulate reactive oxygen species (ROS), including superoxide (O_2_^-^) and hydrogen peroxide (H_2_O_2_) (Zhao et al., 2021). Excessive ROS accumulation can cause oxidative DNA damage, reduce antioxidant capacity, and decrease photosynthesis; in severe cases, it may even induce programmed cell death. To counteract adverse conditions, plants regulate stress-responsive antioxidants (e.g., ascorbic acid and glutathione), metabolites such as proline and glycine betaine, and multiple antioxidant enzymes (e.g., superoxide dismutase (SOD), peroxidase (POD), and catalase (CAT)), thereby facilitating ROS detoxification (Rai et al., 2018). Proline functions as a compatible osmolyte to mitigate abiotic stresses through osmotic adjustment, ROS scavenging, and protection against cell damage (Wang et al., 2023). Beyond oxidative stress, high-temperature stress also damages chloroplasts and mitochondria, leading to degradation of photosynthetic pigments and reduced chlorophyll content, which consequently impairs photosynthesis. High temperatures can readily damage photosystem II (PSII), inhibit photochemical reactions, reduce electron transport efficiency, and hinder energy transfer within the PSII central complex, ultimately causing irreversible plant cell inactivation (Lu and Zhang, 2000; Huan et al., 2014; Yamamoto, 2016). Consequently, photosynthetic rates decline under high-temperature stress, and analyzing these changes can objectively reflect a plant’s thermal adaptation capacity (Marchin et al., 2022). Chlorophyll fluorescence measurements can determine PSII activity, thereby revealing a plant’s response to environmental changes and its photosynthetic mechanisms (Murchie and Lawson, 2013). According to Rochaix (2011), PSII activity can be measured using high-frequency pulse amplitude modulation (PAM) fluorescence emitted by a fluorometer in response to short, intense light pulses.

With the continuous advancement of geographic information systems (GIS), ecological niche modeling (ENM), and species distribution modeling (SDM), statistical modeling has become increasingly sophisticated in ecological research, serving as a key tool for quantifying the complex relationships among species distribution, functional traits, and environmental factors (Escobar and Craft, 2016).Beyond these spatial applications, mathematical modeling combined with empirical data calibration has been widely adopted to solve practical problems in plant stress physiology and functional genomics. For example, untargeted metabolomics combined with multivariate analysis revealed the dynamic changes of phenolic compounds in mung beans during solid-state fermentation (SSF) by Aspergillus niger, identifying 96 phenolic metabolites, of which 61 exhibited significant differential accumulation during fermentation (Lang et al., 2025).

In the context of stress tolerance evaluation, multivariate statistical analysis has proven particularly valuable. Using multivariate statistical analysis of cotton traits under high-temperature stress, Yang et al. (2024) identified two heat-tolerant varieties and established seven physiological indicators as reliable criteria for evaluating heat tolerance in cotton. Similarly, Wang et al. (2022) evaluated the cold tolerance of 20 grape varieties under low-temperature stress using four assessment methods and developed a more accurate screening approach. In an investigation of salt tolerance in asparagus, Gao et al. (2021) identified two salt-tolerant varieties. Collectively, these studies demonstrate that multivariate statistical analysis overcomes the one-sidedness inherent in single-indicator evaluations. To date, however, most methods used to investigate heat tolerance in non-heading Chinese cabbage have been relatively simplistic, and results obtained using different single indicators often vary. Therefore, a comprehensive evaluation system integrating multiple physiological and biochemical indicators is urgently needed for this crop.

To address this gap, the present study selected 35 non-heading Chinese cabbage varieties, measured 13 physiological and biochemical indicators, and employed multivariate methods—including correlation analysis, principal component analysis, membership function analysis, cluster analysis, and stepwise regression analysis—to comprehensively evaluate heat tolerance. In non-heading Chinese cabbage, the seedling stage is the main commercial production stage during the summer season, when high-temperature stress frequently occurs. Therefore, evaluating heat tolerance at the seedling stage is of direct practical importance for summer cultivation and early-season production.

The objectives of this study are fourfold: (1) to elucidate the relationships between individual indicators and heat tolerance; (2) to identify core indicators for heat tolerance evaluation based on physiological and biochemical traits; (3) to develop the Heat Tolerance Productivity Index (HTPI) for quantitative assessment; and (4) to establish a comprehensive evaluation system to streamline the assessment process. The findings can be applied to the selection and breeding of heat-tolerant germplasm and to the standardized evaluation of heat tolerance, thereby supporting year-round vegetable supply and breeding efforts.

## 2 Materials and methods

### 2.1 Plant materials

The test materials comprised 35 non-heading Chinese cabbage varieties provided by the Chinese Cabbage Systems Biology Laboratory at Nanjing Agricultural University. These varieties were designated B1 to B35, with individual names listed in **Table 1**. Based on preliminary screening, B33 was identified as a heat-sensitive variety and was used as the control. The experiment was conducted in July 2023 in Nanjing, Jiangsu Province, China (31°34′ N, 119°09′ E), under conditions suitable for the purposes of this study.

**Table 1:** Table of experimental materials.

Number	Variety	Number	Variety
B1	Huangmeigui	B19	K2223
B2	NJAU08	B20	K2227
B3	Z0123	B21	LBC86
B4	NJAU09	B22	NJAU01
B5	Z0223	B23	F0122
B6	Z0423	B24	Y66
B7	NJAU10	B25	Shiliu
B8	NJAU11	B26	F2222
B9	NJAU12	B27	F4922
B10	Naire 1	B28	F5722
B11	Suqing	B29	F7822
B12	NJAU13	B30	Kangre
B13	NJAUB6	B31	Xiaqing
B14	NJAU14	B32	NJAU16
B15	NJAU15	B33	Aizhuang
B16	Huaguan	B34	LBC97
B17	NJAUA06	B35	Zizuan
B18	NJAUA04		

A completely randomized block design was employed, with three biological replicates per variety. Each plot was surrounded by 2-m buffer rows, and each subplot had an area of 5 m², in which 8.8 g of seeds were broadcast-sown uniformly. Weeding was performed regularly, and no insecticides or plant growth regulators were applied to ensure consistent cultivation conditions and management practices across all plots.

**Figure 1:**
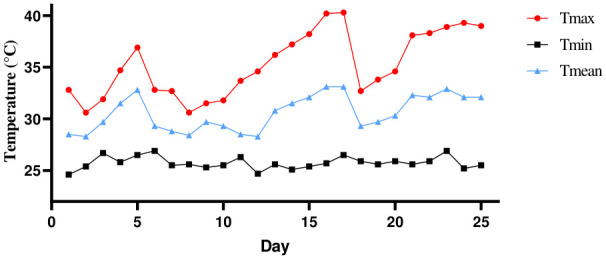
The temperature changes during the growth period of Non-heading Chinese cabbage.

During the experiment, air temperature was recorded daily. A sustained high-temperature stress period occurred from day 11 to day 24, with daily mean temperatures exceeding 30.0 °C and a peak maximum of 41.0 °C on day 17 (detailed temperature dynamics are shown in **Figure 1**). These conditions were well above the optimal growth temperature (18-25 °C) for non-heading Chinese cabbage and constituted an effective heat stress treatment. Thus, the plants experienced heat stress continuously during the seedling stage (from germination to the 3–4 true leaf stage). No heat stress was applied during reproductive stages (flowering or bolting).The experiment was conducted in the field under natural light and ambient humidity conditions.

At maturity, three plants of similar size and growth stage were selected from each experimental plot as three biological replicates. From each selected plant, older leaves were removed, and clean leaves were collected. The leaf material from each plant was processed separately (mixed and chopped) without pooling among plants. Each biological replicate was measured three times (technical replicates), and the average value was used for statistical analysis.

### 2.2 Determination of parameters and methods

Relative Conductivity: Fresh leaf discs were excised, rinsed, and dried. Three replicates of 0.1 g samples were placed in centrifuge tubes containing 10 mL of deionized water and incubated at 25°C for 12 hours. Initial conductivity (R1) was measured using a conductivity meter (Leizhi DDB-303A, China). The samples were then heated in a boiling water bath at 100°C for 20 minutes, cooled to room temperature, and the post-boiling conductivity (R2) was measured. The conductivity of deionized water was recorded as R0. Relative conductivity (REC) was calculated as follows: REC = (R1 – R0)/(R2 – R0) × 100% (Yao et al., 2022).

Relative Chlorophyll Content: Relative chlorophyll content was determined using a SPAD-502 Plus portable chlorophyll meter (KONICA MINOLTA, Japan). Measurements were taken in the early morning of the same day to minimize the influence of solar radiation on SPAD measurement accuracy.

Chlorophyll Fluorescence Parameters: Chlorophyll fluorescence parameters were measured using a modulated chlorophyll fluorescence imaging system (MIMAGING-PAM, Germany). The fourth functional leaf from the base was selected. Each leaf was wrapped in aluminum foil and kept in darkness for 30 minutes prior to measurement. According to Yuan et al. (2014), initial fluorescence F_0_, maximum fluorescence yield F_m_, and the maximum photochemical efficiency of PSII F_v_/F_m_ were measured and calculated. Fluorescence image analysis and quantitative analysis of fluorescence parameters were performed using Imaging Win software (Walz, Effeltrich, Germany). Fluorescence images were captured from the same region on each leaf, with three measurements taken per region, totaling nine measurements per variety, and the final results were averaged.

**Figure 2:**
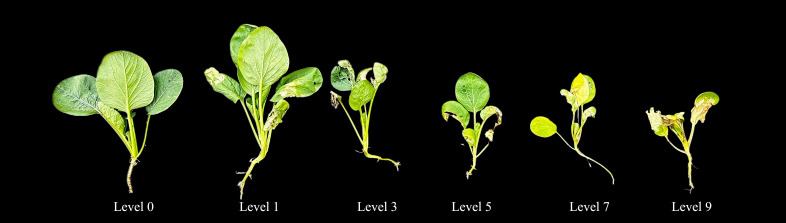
Representative photographs of heat damage levels in non-heading Chinese cabbage according to the 0–9 scoring system.

Heat damage was visually assessed and classified into nine severity levels to define the Heat Damage Index (HDI). The criteria were as follows: Level 0, no visible symptoms, plants growing normally; Level 1, less than one-third of leaves showing damage symptoms; Level 3, approximately one-third of leaves damaged; Level 5, approximately half of leaves damaged; Level 7, approximately two-thirds of leaves damaged but the plant remaining alive; Level 9, plant dead or with no economic value. Representative photographs for damage levels 0, 1, 3, 5, 7 and 9 are provided in **Figure 2**.

The heat damage index (HDI) was computed following the calculation method reported by Dong et al. (2024). Briefly, individual plants were graded on a 0–9 heat injury scale in this experiment, while a 0–5 grading standard was applied in their research. The unified calculation formula is shown below:


$$HDI=\frac{\sum \left(\text{Number of plants at each level}\times \text{Level}\right)}{\text{Highest level}\times \text{Total number of plants surveyed}}\times 100\%$$

Determination of Physiological and Biochemical Parameters: We quantified the enzymatic activities of superoxide dismutase (SOD), catalase (CAT), and peroxidase (POD), alongside the concentrations of superoxide anion (O_2_^-^), hydrogen peroxide (H_2_O_2_), and proline (Pro), were quantified using commercially sourced reagent kits (Comin Biotechnology Co., Ltd., Suzhou, China) (Yang et al., 2020; Weng et al., 2022). The quantity of malondialdehyde (MDA) was assessed following the methodology described by Shah et al. (2001).

### 2.3 Data analysis

Data analysis was performed using Excel and SPSS 26.0 statistical software. Correlation analysis, principal component analysis, and cluster analysis were conducted based on the experimental data, and data visualization was performed using GraphPad Prism 9.5 software.

Analytical framework and model selection. To comprehensively evaluate heat tolerance in non-heading Chinese cabbage, we employed a multi-step analytical pipeline consisting of four complementary components: principal component analysis (PCA), membership function-based HTPI calculation, cluster analysis, and multiple stepwise regression. This combination was selected because it addresses four distinct aspects of heat tolerance evaluation – dimensionality reduction, ranking, classification, and driver identification – which together provide a more robust and informative assessment than any single model.

Specifically, PCA was first applied to reduce the dimensionality of the correlated physiological indicators and to extract independent principal components (PCs) that capture the majority of variance in heat tolerance-related traits, thereby eliminating collinearity. Membership function-based HTPI then converts the multiple PCs into a single, continuous index (0–1), enabling quantitative ranking of varieties. Cluster analysis classifies varieties into discrete heat tolerance categories (e.g., highly tolerant, sensitive), supporting practical breeding recommendations. Multiple stepwise regression identifies which original indicators are most predictive of HTPI and quantifies their effect sizes, providing biological interpretability.

Principal component analysis. PCA was performed on all physiological and biochemical indicators that exhibited significant differences (*p* < 0.05)) to extract principal components and obtain their eigenvalues, variance contribution rates, and cumulative contribution rates.

Calculation of the Heat Tolerance Productivity Index (HTPI).The membership function value for each comprehensive indicator was calculated as follows:


$$\mu \left(X_{i}\right)=\frac{X_{i}-X_{min}}{X_{max}-X_{min}}$$

Where Xi represents the value of the i-th comprehensive indicator, and Xmin and Xmax denote the minimum and maximum values of the comprehensive indicators, respectively.

The weight of each comprehensive indicator was determined based on its contribution rate:


$$W_{i}=\frac{P_{i}}{\sum_{i=1}^{n}P_{i}}$$

Where *W_i_* is the weight of the *i*-th comprehensive indicator, and *P_i_* is the contribution rate of the *i*-th comprehensive indicator.

The comprehensive heat tolerance evaluation score was then calculated as follows:


$$HTPI=\sum_{i=1}^{n}\left[\mu \left(X_{i}\right)\times W_{i}\right]$$

where HTPI (Heat Tolerance Productivity Index) represents the comprehensive heat tolerance evaluation score for different non-heading Chinese cabbage varieties under high-temperature stress, derived from the comprehensive indicators.

Cluster analysis. Cluster analysis was applied to the HTPI values to classify and evaluate the heat tolerance levels of the varieties.

Regression analysis and model validation. Using HTPI as the core dependent variable, multiple stepwise regression analysis was employed to establish an equation for screening and identifying the key indicators influencing heat tolerance in non-heading Chinese cabbage. To assess the risk of overfitting, we performed leave-one-out cross-validation (LOOCV) on the full model. The cross-validated *Q^2^* was 0.998, which was nearly identical to the apparent *R^2^* of 0.999, indicating high model stability and no severe overfitting.

## 3 Results

### 3.1 Temperature dynamics during the experimental period

The daily maximum (Tmax), minimum (Tmin), and mean (Tmean) air temperatures recorded during the experiment are presented in **Figure 1**. The optimal growth temperature for non−heading Chinese cabbage is 18-25 °C. During the early stage of the experiment (days 1-10), the daily mean temperature ranged from 28.5 °C to 30.0 °C, which already exceeded the optimal range. A more severe and prolonged high-temperature stress occurred from day 11 to day 24. During this period, the daily mean temperature consistently remained above 30.0 °C for 12 consecutive days (days 13-24), with a maximum of 33.5 °C recorded on day 17. The daily maximum temperature reached as high as 41.0 °C on day 17, while the daily minimum temperature during the stress phase ranged from 24.5 °C to 27.5 °C providing limited nighttime cooling. These conditions represent a significant and sustained heat stress for non-heading Chinese cabbage, exceeding its optimal temperature range by a substantial margin.

### 3.2 Analysis of differences in heat tolerance indicators of non-heading Chinese cabbage

#### 3.2.1 Analysis of differences in physiological and biochemical indicators

**Figure 3:**
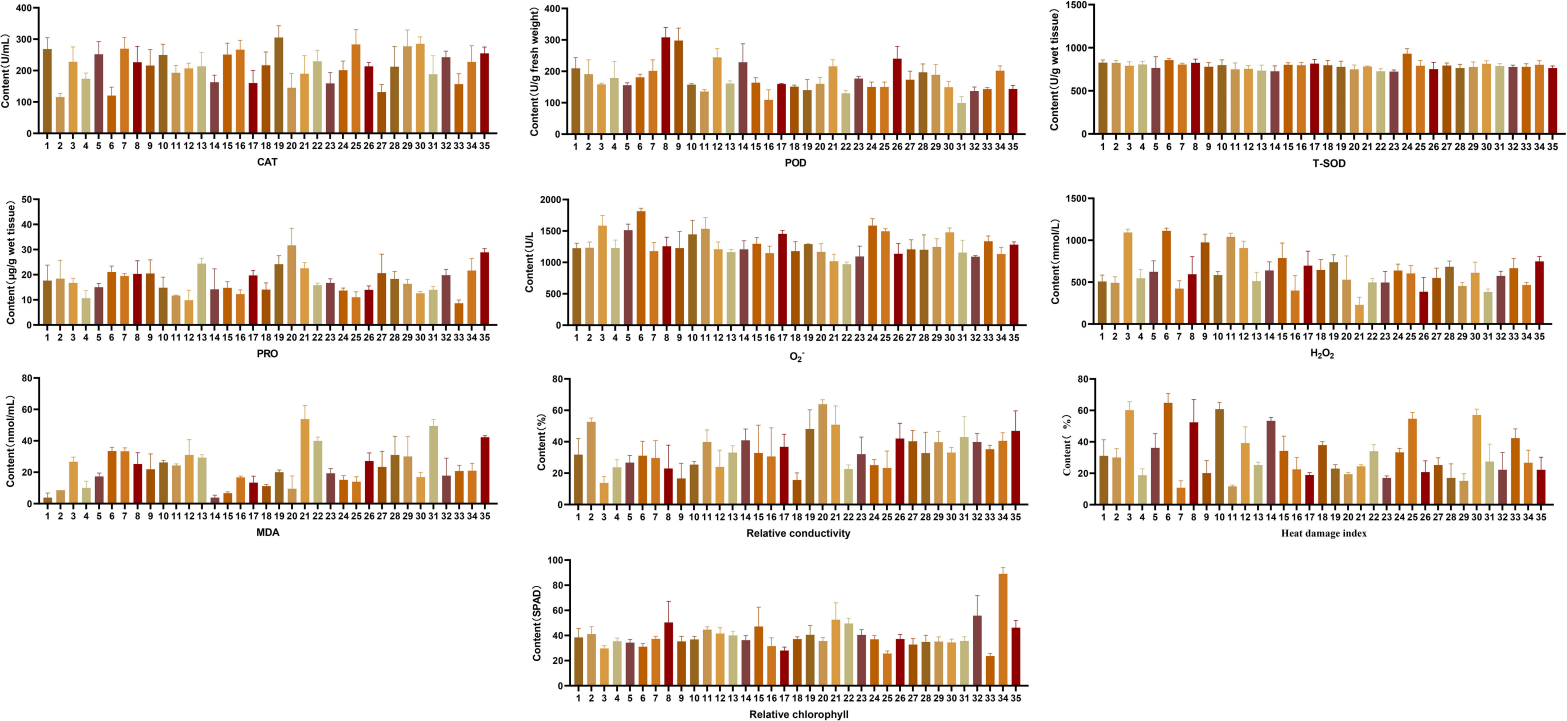
Determination of physiological and biochemical parameters of non-heading Chinese cabbage. Significant differences in the figure are defined at the *p <* 0.05 level.

Analysis of the measured indicators across the 35 non-heading Chinese cabbage varieties revealed significant differences in their responses to high-temperature stress, indicating variations in heat tolerance among the varieties. As depicted in **Figure 3**, the levels of 10 physiological and biochemical indicators are presented for the different varieties.

In studies of high-temperature stress in non-heading Chinese cabbage, physiological and biochemical indicators are generally classified into four main categories: (1) oxidative stress and antioxidant enzyme systems, including O_2_^-^, H_2_O_2_, CAT, POD, SOD; (2) membrane damage and osmotic regulation systems, including Pro, MDA, and REC; (3) morphological damage phenotypic indicators, specifically the HDI; and (4) photosynthetic indicators, including relative chlorophyll content (Chl), initial fluorescence F_0_, F_m_, and F_v_/F_m_.

According to **Figure 3**, substantial variation was observed among the varieties. Compared with the control variety B33, varieties B7 and B10 exhibited higher SOD and CAT activities, yet their heat damage index (HDI) remained relatively high. In contrast, varieties B8 and B9 showed higher POD activity and proline (Pro) content, while their relative electrolyte leakage (REC) remained at a relatively low level. These observations suggest that a single physiological parameter is insufficient to comprehensively reflect a plant’s heat tolerance.

Under high-temperature conditions, variations were observed in the initial fluorescence (F_0_), maximum fluorescence (F_m_), and the maximum photochemical efficiency of PSII (F_v_/F_m_) in the leaves of non-heading Chinese cabbage. As shown in **Table 2**, chlorophyll fluorescence parameters exhibited highly significant differences (*p* < 0.01) among the varieties. The F_v_/F_m_ value of the control variety B33 was 0.623, which was significantly lower than that of healthy plants, indicating that the test materials were substantially affected by high-temperature stress.

**Table 2:** Comparative analysis of chlorophyll fluorescence parameters of no-heading Chinese cabbage.

Variety no.	F_0_	F_m_	F_v_/F_m_
B1	0.098 ± 0.001abcdef	0.316 ± 0.041bcdefghij	0.686 ± 0.045cdefg
B2	0.091 ± 0.001bcdef	0.318 ± 0.007bcdefghi	0.741 ± 0.001abc
B3	0.094 ± 0.004abcdef	0.226 ± 0.009ijk	0.597 ± 0.004kl
B4	0.088 ± 0.001def	0.23 ± 0.001hijk	0.619 ± 0.001ijkl
B5	0.106 ± 0.004abcd	0.218 ± 0.006jk	0.538 ± 0.019m
B6	0.108 ± 0.001ab	0.241 ± 0.009fghijk	0.564 ± 0.017lm
B7	0.091 ± 0.017bcdef	0.294 ± 0.033bcdefghijk	0.693 ± 0.027bcdef
B8	0.096 ± 0.006abcdef	0.347 ± 0.038abcde	0.718 ± 0.042abcde
B9	0.081 ± 0.007f	0.288 ± 0.041bcdefghijk	0.718 ± 0.016abcde
B10	0.093 ± 0.004bcdef	0.281 ± 0.002bcdefghijk	0.67 ± 0.002defghi
B11	0.1 ± 0.005abcde	0.239 ± 0.028ghijk	0.614 ± 0.047ijkl
B12	0.095 ± 0.011abcdef	0.337 ± 0.022abcdef	0.72 ± 0.017abcd
B13	0.103 ± 0.001abcde	0.29 ± 0.005bcdefghijk	0.627 ± 0.021hijk
B14	0.094 ± 0.016abcdef	0.265 ± 0.098defghijk	0.696 ± 0.046abcdef
B15	0.087 ± 0.005ef	0.274 ± 0.024cdefghijk	0.683 ± 0.009cdefgh
B16	0.086 ± 0.008ef	0.294 ± 0.057bcdefghijk	0.703 ± 0.035abcdef
B17	0.087 ± 0.004ef	0.234 ± 0.008hijk	0.632 ± 0.014ghijk
B18	0.094 ± 0.003abcdef	0.282 ± 0.011bcdefghijk	0.659 ± 0.016efghij
B19	0.089 ± 0.008cdef	0.337 ± 0.018abcdefg	0.737 ± 0.017abc
B20	0.093 ± 0.007bcdef	0.347 ± 0.027abcde	0.732 ± 0.007abc
B21	0.104 ± 0.008abcde	0.274 ± 0.034cdefghijk	0.682 ± 0.004cdefgh
B22	0.101 ± 0.003abcde	0.253 ± 0.006efghijk	0.603 ± 0.003jkl
B23	0.101 ± 0.01abcde	0.375 ± 0.037ab	0.73 ± 0.027abcd
B24	0.109 ± 0.001ab	0.273 ± 0.006cdefghijk	0.605 ± 0.01jkl
B25	0.106 ± 0.005abcd	0.271 ± 0.006defghijk	0.606 ± 0.007jkl
B26	0.093 ± 0.006bcdef	0.327 ± 0.049abcdefgh	0.713 ± 0.043abcde
B27	0.1 ± 0.012abcde	0.348 ± 0.083abcde	0.691 ± 0.037cdef
B28	0.112 ± 0.008a	0.338 ± 0.099abcdef	0.707 ± 0.04abcde
B29	0.103 ± 0.006abcde	0.37 ± 0.04abc	0.72 ± 0.032abcd
B30	0.102 ± 0.004abcde	0.199 ± 0.001k	0.623 ± 0.011ijk
B31	0.087 ± 0.002ef	0.353 ± 0.007abcd	0.752 ± 0.004ab
B32	0.107 ± 0.009abc	0.417 ± 0.048a	0.742 ± 0.008abc
B33	0.103 ± 0.005abcde	0.239 ± 0.004ghijk	0.623 ± 0.007ijk
B34	0.088 ± 0.008def	0.309 ± 0.042bcdefghij	0.755 ± 0.015a
B35	0.09 ± 0.001cdef	0.248 ± 0.008fghijk	0.647 ± 0.005fghijk

Mean ± SD. Values followed by different letters mean significance of difference between the treatments (*p* < 0.01).

Among the test materials, B34 exhibited the highest F_v_/F_m_ value, which was significantly higher than that of most other varieties, whereas B5 showed the lowest F_v_/F_m_ value, representing only 71.3% of that of B34. These results suggest that B34 maintained a high light energy conversion efficiency under high-temperature stress, while the photosynthetic system of B5 was the most severely impaired. Substantial variation among varieties was also observed in F_0_ and F_m_ values, with B32 exhibiting the highest F_0_ value and B30 the lowest, further confirming differences in photosynthetic system responses to high-temperature stress.

Overall, these results indicate that under high-temperature stress, the initial light energy conversion efficiency of PSII decreases, leading to a significant reduction in the F_v_/F_m_ ratio, which consequently affects plant photosynthesis. This further validates the differential responses of non-heading Chinese cabbage varieties to high-temperature stress.

#### 3.2.2 Correlation analysis

**Figure 4:**
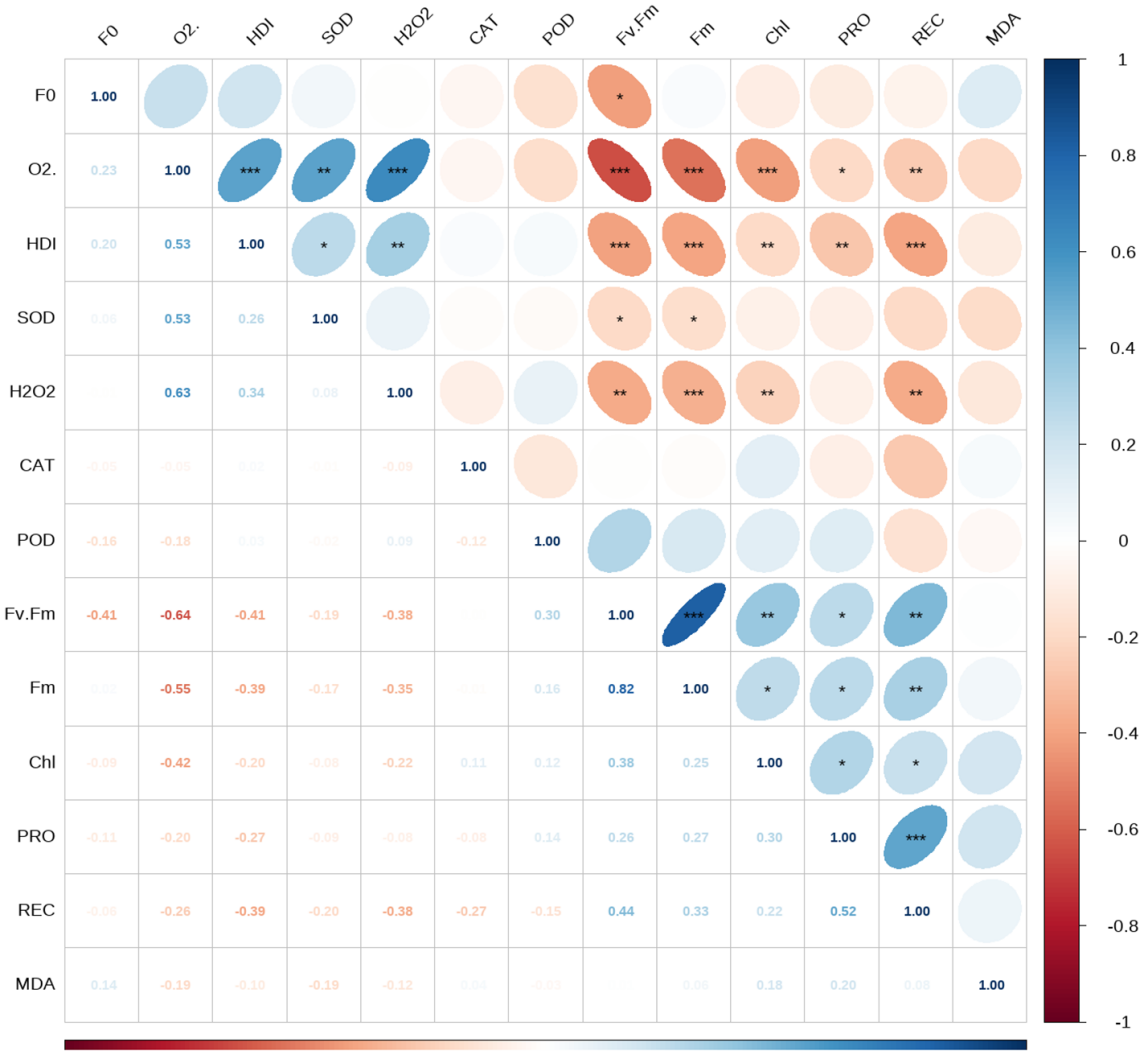
Correlation coefficient matrix of each single index. Pearson’s correlation coefficients are shown for initial fluorescence (F_0_), superoxide anion radical (O_2_^-^), heat damage index (HDI), superoxide dismutase (SOD), hydrogen peroxide (H_2_O_2_), catalase (CAT), peroxidase (POD), maximum photochemical efficiency of PSII (F_v_/F_m_), maximum fluorescence (F_m_), relative chlorophyll content (Chl), proline (Pro), relative electrical conductivity (REC), and malondialdehyde (MDA). Asterisks denote significance levels (*p< 0.05, **p < 0.01, ***p < 0.001).

Correlation analysis was performed on the 13 individual indicators of non-heading Chinese cabbage (**Figure 4**). Considerable variation was observed in the physiological and biochemical indicators among different varieties, indicating that the heat tolerance coefficient based on a single indicator cannot comprehensively and accurately reflect the overall level of heat tolerance. As shown in the correlation matrix, complex linear relationships exist among the various indicators. Therefore, systematic analysis of these correlations is essential for establishing a comprehensive evaluation system for heat tolerance.

As shown in **Figure 4**, SOD content was highly significantly positively correlated with O_2_^-^ content (0.531), indicating that higher SOD content was associated with higher O_2_^-^ content. Pro content showed a highly significant positive correlation with REC (0.520), suggesting that higher Pro content corresponded to higher REC values. F_v_/F_m_ exhibited negative correlations with HDI (−0.408) and O_2_^-^ content (−0.641), indicating that higher F_v_/F_m_ values were associated with lower HDI and O_2_^-^content. F_m_ showed a highly significant positive correlation with F_v_/F_m_ (0.819) and a highly significant negative correlation with O_2_^-^ content (−0.548); that is, higher F_m_ values were associated with increased F_v_/F_m_ values and decreased O_2_^-^ content.

In summary, information overlap exists among the various heat tolerance evaluation indices for non−heading Chinese cabbage, making it difficult for any single index to independently and accurately reflect heat tolerance. This indicates that heat tolerance in non−heading Chinese cabbage is a complex quantitative trait regulated by multiple indices, and evaluation based on a single index alone is insufficient. Therefore, multivariate statistical methods such as principal component analysis and cluster analysis should be further employed to transform multiple related indices into a few highly independent composite indicators, thereby establishing a more scientific and objective comprehensive evaluation system for heat tolerance.

#### 3.2.3 Principal component analysis

Principal component analysis was performed on the individual indicators of the 35 non-heading Chinese cabbage varieties. The number of principal components to be extracted was determined based on a comprehensive evaluation of the Kaiser criterion (eigenvalue > 1) and the inflection point principle, which identifies the point on the screen plot where the slope of eigenvalue decline becomes distinctly flattened. The analysis was truncated at a cumulative contribution rate of 71.3%, yielding six independent principal components that effectively represent the majority of the information contained in the original indicators. As shown in **Table 3**, the contribution rates of the six principal components are 25.739%, 10.442%, 9.477%, 9.050%, 8.568%, and 8.025%, respectively.

**Table 3:** Eigenvalue, contribution rate and feature vector of each comprehensive indicator of non-heading Chinese cabbage.

Variable	CI1	CI2	CI3	CI4	CI5	CI6
Eigenvalues	3.346	1.357	1.232	1.176	1.114	1.043
Contribution rate	25.739	10.442	9.477	9.050	8.568	8.025
Cumulative contribution rate	25.739	36.180	45.658	54.707	63.275	71.300
SOD	0.061	0.115	-0.014	0.024	0.787	0.104
POD	-0.040	0.030	0.682	0.012	-0.001	-0.045
Pro	-0.132	0.560	0.017	0.010	0.093	-0.141
Chl	-0.074	0.421	0.149	-0.042	0.017	0.333
REC	0.148	0.236	-0.308	-0.019	0.177	-0.324
F0	0.102	-0.068	0.010	0.716	0.094	-0.050
Fm	0.363	-0.160	0.234	0.241	0.084	-0.085
Fv/Fm	0.289	-0.042	0.210	-0.121	0.099	-0.032
HDI	-0.109	-0.158	0.199	0.148	0.019	-0.011
MDA	-0.086	0.296	0.014	0.428	-0.323	0.132
CAT	0.052	0.005	-0.088	-0.007	0.098	0.742
H2O2	-0.364	0.136	0.261	-0.122	-0.074	-0.146
O_2_^-^	-0.206	0.031	0.018	0.089	0.286	-0.083

Based on the analysis of the component matrix, the six principal components (CIs) were characterized as follows: CI1 was predominantly composed of REC, F_m_, F_v_/F_m_, and H_2_O_2_; CI2 consisted mainly of Pro, Chl, and MDA; CI3 comprised POD, REC, F_m_, H_2_O_2_, and HDI; CI4 was primarily defined by F_0_, F_m_, and MDA; CI5 consisted of SOD, MDA, and O_2_^-^; and CI6 was composed of Chl, REC, and CAT.

### 3.3 Comprehensive evaluation of heat tolerance in non-heading Chinese cabbage

#### 3.3.1 Membership function analysis and comprehensive evaluation

The membership function method from fuzzy mathematics was employed to quantify and integrate the membership degrees of various heat tolerance indicators for non-heading Chinese cabbage. The Heat Tolerance Productivity Index (HTPI) was subsequently calculated, with higher HTPI values indicating superior heat tolerance performance.

The membership function values for the six principal components were calculated based on the weights derived from principal component analysis. These values varied considerably across the tested varieties. For instance, B6 exhibited the highest membership degree in PC3 (0.977) but the lowest in PC1 (0.047). B35 showed the lowest membership value in PC5 (0.063), whereas B21 displayed the highest membership value in PC2 (0.980).

According to **Table 4**, the weights of the six comprehensive indicators for non-heading Chinese cabbage were 0.361, 0.146, 0.133, 0.127, 0.120, and 0.113, respectively. Since a higher HTPI value indicates stronger heat tolerance, the varieties were ranked in ascending order of HTPI as follows: B3< B4< B17< B33< B5< B6< B30< B25< B11< B9< B18< B14< B15< B10< B12< B16< B24< B1< B22< B2< B35< B13< B7< B26< B19< B8< B27< B23< B28< B31< B20< B29< B34< B32< B21. Accordingly, the five varieties with the highest heat tolerance were B21, B32, B34, B29, and B20, whereas the five with the lowest heat tolerance were B3, B4, B17, B33, and B5.

**Table 4:** Comprehensive index values of no-heading Chinese cabbage.

Number	μ(X1)	μ(X2)	μ(X3)	μ(X4)	μ(X5)	μ(X6)	HTPI-value	Order
B1	0.577	0.357	0.447	0.610	0.577	0.680	0.543	18
B2	0.723	0.493	0.587	0.180	0.627	0.597	0.577	16
B3	0.145	0.550	0.555	0.605	0.315	0.300	0.355	35
B4	0.433	0.457	0.280	0.397	0.400	0.477	0.413	34
B5	0.213	0.773	0.367	0.660	0.473	0.483	0.434	31
B6	0.047	0.807	0.977	0.450	0.590	0.400	0.438	30
B7	0.693	0.583	0.400	0.713	0.397	0.560	0.590	13
B8	0.637	0.230	0.820	0.833	0.583	0.400	0.594	10
B9	0.590	0.157	0.680	0.557	0.230	0.210	0.448	26
B10	0.387	0.517	0.420	0.693	0.517	0.503	0.478	22
B11	0.357	0.790	0.470	0.460	0.313	0.387	0.446	27
B12	0.563	0.320	0.550	0.717	0.583	0.167	0.503	21
B13	0.607	0.873	0.467	0.643	0.467	0.373	0.588	14
B14	0.530	0.380	0.460	0.347	0.580	0.253	0.450	24
B15	0.503	0.370	0.403	0.510	0.283	0.590	0.455	23
B16	0.607	0.523	0.077	0.527	0.487	0.657	0.505	20
B17	0.413	0.617	0.483	0.203	0.347	0.553	0.433	33
B18	0.440	0.430	0.317	0.580	0.493	0.463	0.449	25
B19	0.720	0.607	0.450	0.550	0.317	0.673	0.592	11
B20	0.870	0.773	0.610	0.110	0.517	0.537	0.645	5
B21	0.840	0.980	0.630	0.787	0.487	0.447	0.739	1
B22	0.550	0.887	0.267	0.850	0.370	0.280	0.547	17
B23	0.775	0.635	0.390	0.450	0.735	0.305	0.604	8
B24	0.240	0.610	0.607	0.587	0.703	0.857	0.512	19
B25	0.223	0.563	0.297	0.710	0.710	0.543	0.439	28
B26	0.763	0.503	0.403	0.580	0.610	0.373	0.592	12
B27	0.667	0.693	0.573	0.370	0.720	0.440	0.601	9
B28	0.643	0.707	0.553	0.687	0.740	0.370	0.627	7
B29	0.730	0.630	0.403	0.740	0.720	0.560	0.653	4
B30	0.240	0.610	0.357	0.670	0.480	0.640	0.438	29
B31	0.780	0.803	0.283	0.543	0.620	0.493	0.636	6
B32	0.830	0.660	0.500	0.760	0.750	0.600	0.717	2
B33	0.307	0.703	0.323	0.390	0.713	0.373	0.434	32
B34	0.913	0.473	0.670	0.783	0.107	0.657	0.674	3
B35	0.630	0.950	0.590	0.610	0.063	0.503	0.587	15
Weight	0.361	0.146	0.133	0.127	0.120	0.113		

#### 3.3.2 Cluster analysis

Hierarchical cluster analysis was performed on the indicators of non-heading Chinese cabbage using the comprehensive Heat Tolerance Productivity Index (HTPI) as the input variable and Euclidean distance as the similarity metric, with the Ward method applied for agglomeration. Based on a distance threshold of 0.15, the heat tolerance of the tested materials was classified into four levels.

**Figure 5:**
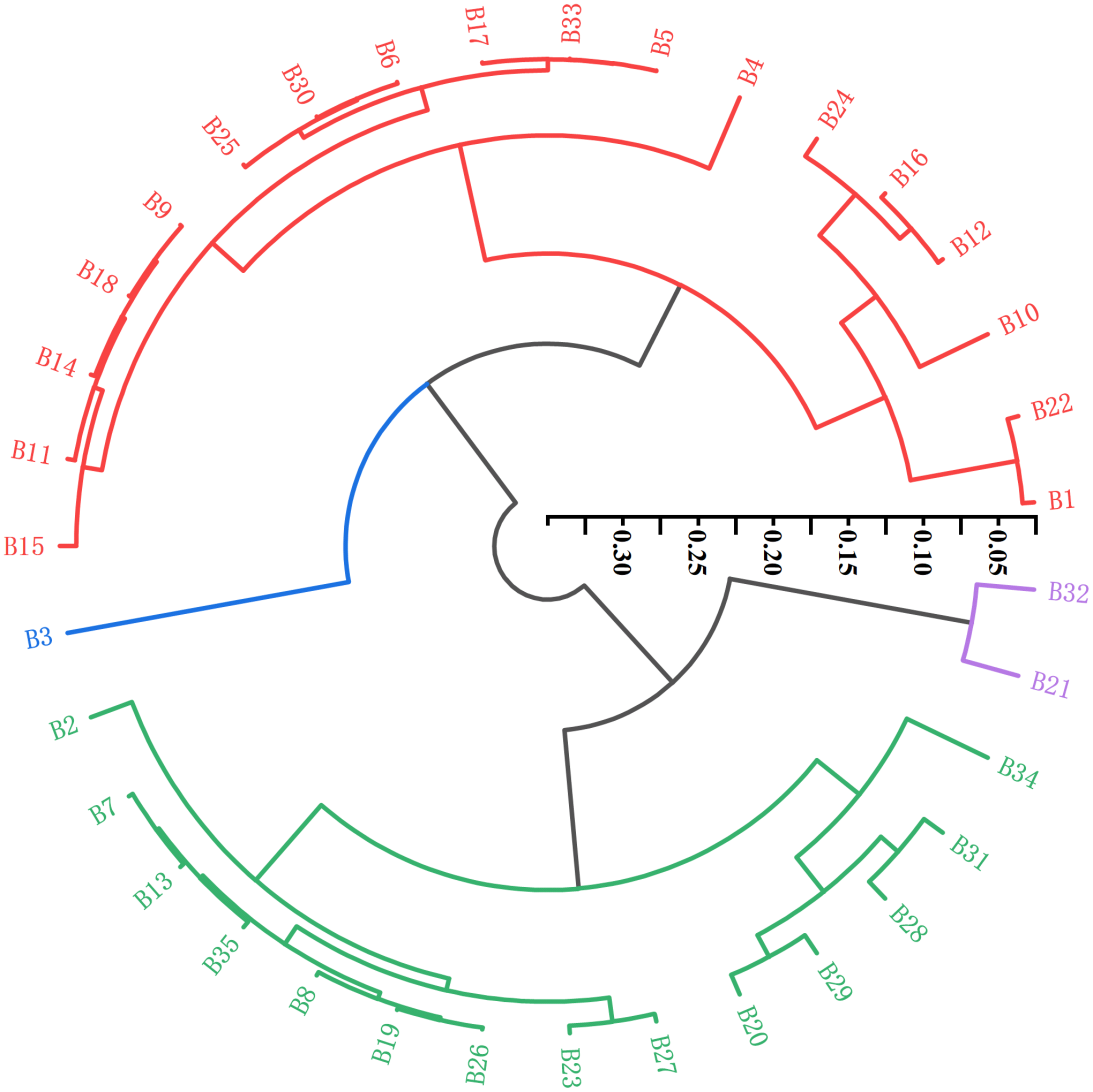
Hierarchical clustering of heat tolerance in non-heading Chinese cabbage.

**Figure 6:**
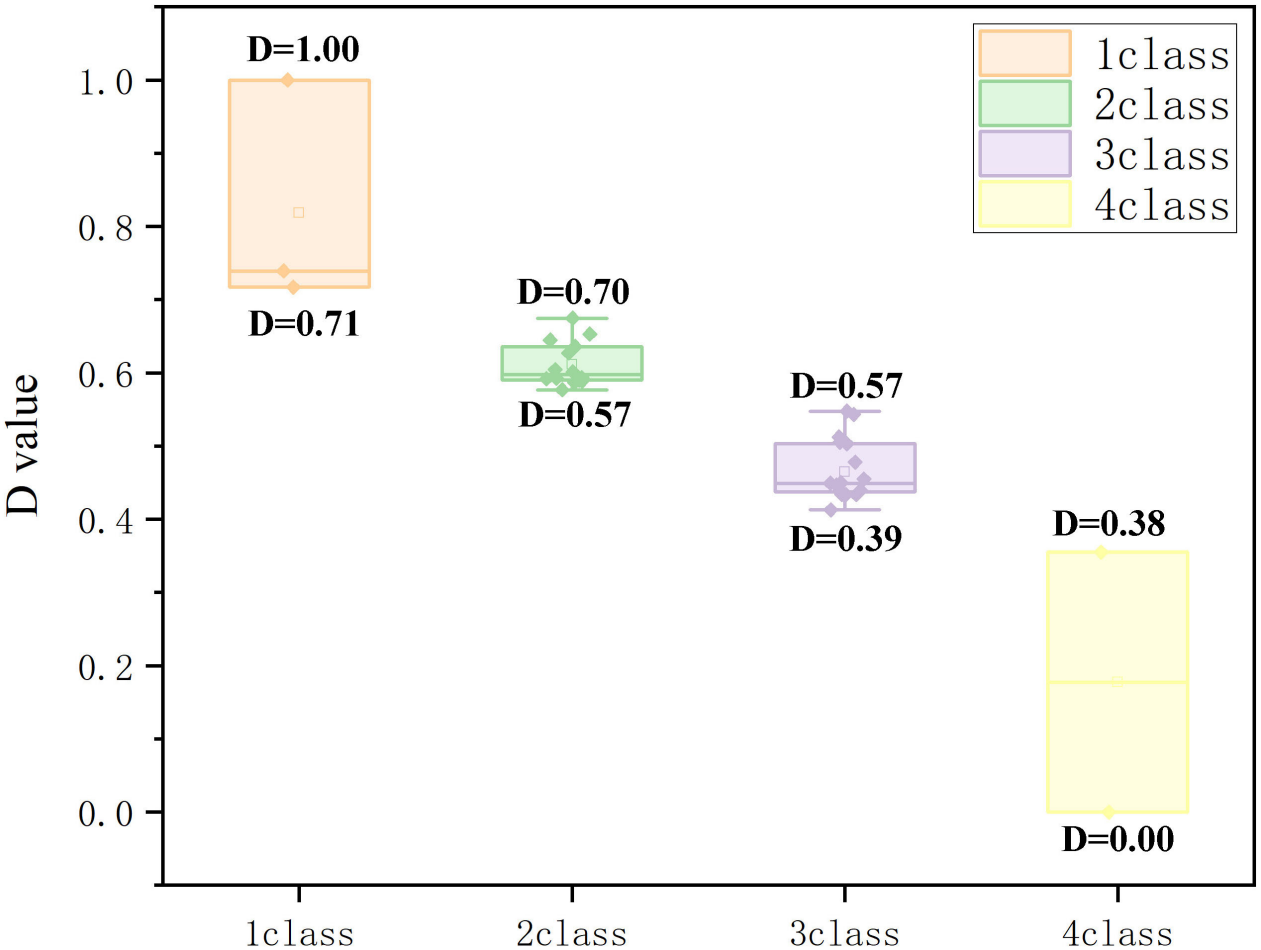
Interval plot of heat tolerance grades in non-heading Chinese cabbage.

Based on the clustering results (**Figure 5**), the 35 varieties were classified into four categories: Class I (highly heat-tolerant) comprised two varieties (B21 and B32); Class II (moderately heat-tolerant) comprised 14 accessions; Class III (heat-sensitive) comprised 18 accessions; and Class IV (extremely heat-sensitive) comprised one accession (B3). Accordingly, HTPI ranges were defined as follows: highly heat-tolerant, 0.71–1; moderately heat-tolerant, 0.57–0.70; heat-sensitive, 0.39–0.57; and extremely heat-sensitive, 0–0.38 (**Figure 6**).

#### 3.3.3 Screening of heat tolerance evaluation indices

Regression analysis was conducted to establish a mathematical model for heat tolerance evaluation, with the heat tolerance coefficients of individual indicators as independent variables and the comprehensive Heat Tolerance Productivity Index (HTPI) as the dependent variable. The optimal regression equation for heat tolerance in non-heading Chinese cabbage was derived as follows:HTPI=-0.358 + 0.567F_m_-0.000093H_2_O_2_+0.003Pro+0.002MDA+0.002Chl+0.002REC+ 2.877F_0_-0.001HDI+0.211F_V_/F_m_-0.000066O_2_^-^+0.000038POD.

The established regression model demonstrated an excellent goodness of fit, indicating that the independent variables explained 99.9% of the variance in the dependent variable (*R²* = 0.999). Analysis of variance (ANOVA) revealed that the model was highly significant (*F* = 2430.061, *p* < 0.001). The Durbin-Watson statistic was 1.918, suggesting no significant autocorrelation in the residuals. Collectively, these results demonstrate that the regression model possesses strong explanatory power and reliability.

From the individual indicators, 11 key indices were selected: F_m_, H_2_O_2_, Pro, MDA, Chl, REC, F_0_, HDI, F_v_/F_m_, O_2_^-^, and POD. These indicators were significantly correlated with heat tolerance in non-heading Chinese cabbage and can therefore be used to evaluate heat tolerance in this crop.

To further assess the relative contribution of each physiological indicator to the heat tolerance prediction model, LASSO regression was performed. The variable importance ranking based on the absolute coefficients of LASSO regression is presented in **Table 5**. The three most influential variables were F_0_, F_m_, and F_v_/F_m_, together accounting for 99.72% of the total absolute coefficient sum (**Table 5**). Although these three variables had the highest weights, attempts to construct a reduced model using only them resulted in a substantial drop in predictive performance (*R^2^* = 0.65, *Q^2^* = 0.53), indicating that the remaining indicators, despite their smaller coefficients, collectively contribute essential information for accurate HTPI prediction.

**Table 5:** Variable importance ranking based on LASSO regression coefficients.

Rank	Variable	Coefficient	Cumulative (%)
1	F_0_	2.663	76.93
2	F_m_	0.591	93.99
3	F_v_/F_m_	0.198	99.72
4	Pro	0.003	99.82
5	MDA	0.002	99.87

Cumulative contribution is calculated as the sum of absolute coefficients of top-ranked variables divided by the total sum of absolute coefficients of all variables. This reflects the relative weight of each variable in the LASSO model, not the proportion of explained variance (R^2^).

## 4 Discussion

High temperature has become a key factor limiting the quality and yield of non-heading Chinese cabbage, significantly affecting its growth and development. Most varieties are highly sensitive to high temperatures, which restricts their cultivation under such conditions. In the context of global warming, breeding and utilization of heat-tolerant varieties have become a key strategy for ensuring stable production. Consequently, establishing a comprehensive evaluation system that integrates multiple physiological and biochemical indicators is essential for assessing heat tolerance. Furthermore, rapid identification of heat tolerance at the early growth stage is of great significance for shortening the breeding cycle and improving breeding efficiency.

### 4.1 Correlation between physiological indicators and heat tolerance

Previous studies have shown that although phenotypic-based identification methods are efficient and straightforward, relying solely on phenotypic traits to evaluate heat tolerance in seedlings has certain limitations. Therefore, accurate assessment of heat tolerance in Chinese cabbage necessitates the integration of phenotypic observations with physiological and biochemical indicators to establish a comprehensive multi-indicator evaluation system, thereby improving the reliability and scientific validity of the results (Shi et al., 2023).

PCA transforms multiple individual indicators into several independent composite indicators, enabling comprehensive evaluation through score calculation (Greenacre et al., 2022). In this study, PCA was employed to reduce 13 individual indicators to six principal components. Subsequently, membership function analysis was applied to derive a comprehensive heat tolerance productivity index (HTPI) for non-heading Chinese cabbage, allowing comparison of heat tolerance levels. Cluster analysis further classified the 35 varieties into four categories: highly heat-tolerant, moderately heat-tolerant, heat-sensitive, and extremely heat-sensitive.

Given that heat tolerance in non-heading Chinese cabbage is governed by multiple independent indicators, the selection of appropriate indicators is critical for its evaluation. Analyzing changes in physiological and biochemical parameters enables assessment of the plant’s osmotic regulation and cellular antioxidant defense capacity under high-temperature stress.

Experiments revealed that Pro content in variety B21 increased significantly under high-temperature stress, indicating an osmotic regulation response. As a key osmotic regulator, Pro accumulation helps maintain cellular homeostasis under high temperature. Moreover, Pro contributes to the maintenance of cellular metabolic function by protecting enzyme activity and stabilizing membrane structure (Bita and Gerats, 2013; Yu et al., 2023; Tang et al., 2025). Thus, B21 establishes a synergistic defense system combining osmotic and macromolecular protection through Pro regulation, which may serve as a critical physiological basis for its strong heat tolerance—a finding consistent with that of Wang et al. (2023).

In plant responses to abiotic stress, ROS act as key signaling components involved in the cross-regulation of multiple stress signaling pathways. Changes in the activity of antioxidant enzyme systems (e.g., CAT, SOD) influence intracellular ROS homeostasis. Plants possess an antioxidant enzyme scavenging system that plays a central role in maintaining the dynamic balance between ROS production and elimination (Alvarez et al., 1998; Medina et al., 2021). Analysis of antioxidant enzyme activity in the tested varieties revealed significant physiological differentiation among varieties with different heat tolerance levels in response to high-temperature stress. Notably, the CAT, SOD, and POD activities of moderately heat-tolerant varieties were generally higher than those of extremely heat-sensitive ones, suggesting a more efficient ROS scavenging system. These results corroborate previous findings in various crops (Gill and Tuteja, 2010; Medina et al., 2021), further supporting the critical role of the antioxidant enzyme system in plant heat tolerance.

Cell membranes are particularly sensitive to high-temperature stress, with lipid peroxidation serving as a key indicator of membrane damage. MDA, the primary product of membrane lipid peroxidation, is commonly used as an indicator of membrane damage severity (Tsikas, 2017). In this study, non-heading Chinese cabbage seedlings exhibited elevated MDA and ROS levels under high-temperature stress, indicating that cells suffered severe damage under thermal stress.

Quan et al. (2022) found that high temperatures may significantly alter the metabolite profile and antioxidant synthesis in Chinese cabbage, ultimately affecting its photosynthetic efficiency and osmoprotective mechanisms. Chlorophyll fluorescence is considered a key indicator of the photosynthetic energy conversion efficiency of PSII; it not only reflects the photochemical activity of PSII but is also sensitive to various stress factors, and is therefore widely used in analyzing plant photosynthetic performance under abiotic stress (Marečková et al., 2019). Jiao and Hu (2025) reported that F_v_/F_m_ in healthy plants typically ranges from 0.8 to 0.85. In this study, F_v_/F_m_ values of the tested varieties under high-temperature stress were all below 0.75, indicating that all were subjected to heat stress.

Correlation analysis revealed negative correlations between F_m_ and HDI (r = −0.39) and between F_v_/F_m_ and HDI (r = −0.41). These results are consistent with Haque et al. (2014), who showed that F_v_/F_m_ decreases significantly under high-temperature stress in wheat, with heat-tolerant varieties exhibiting a smaller decline than heat-sensitive ones. This study further demonstrates that varieties with lower HDI under high-temperature stress suffer more severe damage to the PSII system. Similarly, Prasertthai et al. (2022) found that heat-tolerant rice genotypes maintain greater F_v_/F_m_ stability under high-temperature stress, attributed to the optimization of thylakoid membrane fluidity through adjustments in fatty acid composition, thereby preserving PSII complex integrity at higher temperatures. Additionally, Gomez-Campo and Baums (2025) identified F_v_/F_m_ as a sensitive indicator for quantifying PSII functional loss under heat stress.

Thus, F_m_, F_v_/F_m_, and F_0_ can serve as effective indicators for evaluating heat tolerance in non-heading Chinese cabbage. Under high-temperature stress, strongly heat-tolerant varieties maintain higher F_v_/F_m_ values, reflecting greater light energy conversion efficiency. Collectively, these results reveal germplasm-specific responses to high-temperature stress among different Chinese cabbage varieties, with physiological indicators exhibiting diverse response patterns across the tested materials.

### 4.2 Analysis of the applicability of the comprehensive heat tolerance evaluation model

It is worth noting that although multivariate statistical methods have been applied to evaluate crop heat tolerance, few studies have established simple and effective methods for rapid identification of heat-tolerant varieties. Guo et al. (2025) successfully distinguished the heat tolerance levels of eight rhododendron groups, providing a reference for preliminary screening of heat-tolerant rhododendron germplasm. However, that study did not derive a regression equation to quantify heat tolerance and lacked the ability to identify unknown samples, which to some extent limits the application of its evaluation system across a wide range of rhododendron germplasm resources. Therefore, constructing a comprehensive evaluation model that is applicable to diverse germplasm resources and possesses predictive capability is particularly important.

As stated by Yang et al. (2024), an optimal regression equation for cotton heat tolerance was derived from a study of 21 cotton accessions:D=-3.152 + 0.168Pn+0.382LSOD+2.726Fv/Fm+0.164Φ_PSII_+0.120 LMDA + 0.026LCAT+0.110SDW+0.063RMDA(*R^2^* = 0.987). Although this method allows rapid assessment of heat tolerance in different cotton varieties, its applicability is limited by the relatively small sample size. In the present study, we increased the sample size and expanded the scope of comprehensive heat tolerance evaluation by measuring 13 individual indicators across 35 non-heading Chinese cabbage varieties.

According to Jang et al. (2024), drought tolerance was compared among 100 Chinese cabbage varieties, leading to the selection of two drought−tolerant varieties and the identification of several assessment indicators, including visual wilting score, relative leaf water content, SPAD, and F_v_/F_m_. However, due to the lack of a comprehensive evaluation system for drought tolerance in Chinese cabbage, they were unable to quantify the effects of drought stress on its physiological and biochemical processes.

This study employed multivariate statistical analysis to construct a comprehensive model for evaluating heat tolerance in non-heading Chinese cabbage. By analyzing photochemical reaction efficiency, antioxidant capacity, and osmotic regulation ability under high-temperature conditions, the heat tolerance of 35 varieties was evaluated, and a systematic evaluation model was established. This model quantifies the physiological and biochemical responses underlying heat stress tolerance in non-heading Chinese cabbage.

The Heat Tolerance Productivity Index (HTPI) was developed as a comprehensive evaluation metric, defined as follows:HTPI=-0.358 + 0.567F_m_-0.000093H__2__O__2__+0.003Pro+0.002MDA+0.002Chl+0.002 REC + 2.877F_0_-0.001HDI+0.211F_v_/F_m_-0.000066O_2_^-^+0.000038POD.Using this model, heat tolerance in non-heading Chinese cabbage can be classified, enabling rapid screening of heat-tolerant non-heading Chinese cabbage varieties suitable for high-temperature conditions, thereby supporting year-round supply. The model demonstrated superior discriminatory accuracy, allowing rapid identification of different heat-tolerant germplasm types and precise classification of heat tolerance levels.

Multivariate statistical analysis also facilitates the identification of parental lines in breeding programs. By integrating agronomic, physiological, and heat tolerance traits, this comprehensive evaluation supports early screening of heat-tolerant varieties, informing future field evaluations and breeding decisions in vegetable production systems under global warming. Collectively, the established model provides a critical reference and mathematical tool for the screening, identification, and classification of heat-tolerant germplasm.

Beyond variety screening and parental selection, this evaluation system also provides a reliable platform for identifying key regulatory genes underlying heat tolerance. For instance, the ramie gene *BnWOX14*, which is specifically expressed in stems and roots and positively regulates adventitious root development, could serve as a candidate for root-based heat tolerance improvement (Abubakar et al., 2023). Similarly, the temperature stress-related *qCTG-8* and *qCTG-11* identified in wild rice offer valuable genetic loci for enhancing crop thermotolerance (Pan et al., 2023). Thus, our integrated system holds significant potential for facilitating the discovery and functional characterization of heat-tolerant genes in future breeding efforts.

In addition, this study focused on evaluating heat tolerance in non-heading Chinese cabbage at the seedling stage and did not cover reproductive growth stages, such as flowering and bolting, which are more sensitive to high-temperature stress. Future research should expand the evaluation framework by incorporating additional indicators across multiple growth stages, thereby refining model coverage and broadening its applicability.

Although the final model retained 11 out of 13 initial indicators, this complexity reflects the multifaceted nature of heat tolerance in non-heading Chinese cabbage. Heat tolerance involves coordinated responses of photosynthetic efficiency (F_0_, F_m_, F_v_/F_m_, Chl), antioxidant defense (SOD, POD, CAT), membrane integrity (MDA, REC), and osmotic adjustment (PRO, HDI, H_2_O_2_, O_2_^-^). Removing any of these categories would lose biologically relevant information. Importantly, leave-one-out cross-validation (LOOCV) yielded a *Q^2^* of 0.998, nearly identical to the apparent *R^2^* of 0.999, demonstrating that the excellent fit is not an artifact of overfitting. Thus, while the model is not maximally parsimonious, it is well-justified biologically and statistically robust. For readers who prefer a simpler model, the variable importance ranking (**Table 5**) provides guidance on the most influential indicators.

## 5 Conclusion

Given that heat tolerance in non-heading Chinese cabbage is regulated by multiple factors, a comprehensive evaluation was conducted using multivariate statistical methods, including correlation analysis, principal component analysis, cluster analysis, and regression analysis.

Correlation analysis revealed significant or highly significant correlations among certain physiological and biochemical indicators, indicating synergistic or antagonistic relationships in response to high-temperature stress, with relatively high correlation coefficients. Through PCA and cluster analysis of heat tolerance indicators, the heat tolerance of different varieties was ranked based on the comprehensive HTPI. Cluster analysis further classified the varieties into four heat tolerance groups: highly heat-tolerant, moderately heat-tolerant, heat-sensitive, and extremely heat-sensitive.

A mathematical model for evaluating heat tolerance was subsequently established, from which 11 key indicators, namely F_m_, H_2_O_2_, Pro, MDA, Chl, REC, F_0_, HDI, F_v_/F_m_, O_2_^-^, and POD, were identified as effective parameters for assessing heat tolerance in non-heading Chinese cabbage. Based on this comprehensive evaluation, two highly heat-tolerant varieties (B21 and B32) and one extremely heat-sensitive variety (B3) were identified.

In summary, the evaluation model developed in this study enables rapid identification of heat tolerance in non-heading Chinese cabbage and allows refined classification of heat tolerance levels through quantification of the HTPI. Thus, this model provides a reliable and efficient technical approach for screening and evaluating heat-tolerant germplasm, and offers a valuable reference for future breeding programs and germplasm identification. These findings are of significant importance for future research on heat stress and for predicting market demand trends for non-heading Chinese cabbage.

Limitations and future perspectives. The regression model for HTPI was developed based on 35 varieties and 13 physiological indicators. Although internal validation using leave-one-out cross-validation demonstrated excellent predictive stability (*Q^2^* = 0.998), external validation with an independent set of varieties would further strengthen the generalizability of the model. Future studies with larger and more diverse germplasm collections are warranted to validate and potentially refine the current model.

## Data Availability

The original contributions presented in the study are included in the article and supplementary material; further inquiries can be directed to the corresponding authors.
